# Does an Association among Sarcopenia and Metabolic Risk Factors Exist in People Older Than 65 Years? A Systematic Review and Meta-Analysis of Observational Studies

**DOI:** 10.3390/life13030648

**Published:** 2023-02-26

**Authors:** María del Carmen Carcelén-Fraile, Agustín Aibar-Almazán, Diego Fernando Afanador-Restrepo, Yulieth Rivas-Campo, Carlos Rodríguez-López, María del Mar Carcelén-Fraile, Yolanda Castellote-Caballero, Fidel Hita-Contreras

**Affiliations:** 1 Department of Health Sciences, Faculty of Health Sciences, University of Jaén, 23071 Jaen, Spain; 2 Faculty of Health Sciences and Sport, University Foundation of the Área Andina–Pereira, Pereira 660004, Colombia; 3 Faculty of Human and Social Sciences, University of San Buenaventura-Cali, Santiago de Cali 760016, Colombia; 4 Lecturer University Schools Gimbernat, University of Cantabria, 39005 Santander, Spain

**Keywords:** sarcopenic, metabolic diseases, older adults, systematic review

## Abstract

Sarcopenia is defined as the generalized and progressive loss of skeletal muscle strength and mass that may be affected by metabolic factors, although this relationship has been poorly studied. The aim of this review and meta-analysis was to analyze the relationship among the different metabolic risk factors and sarcopenia in people older than 65 years. Following the PRISMA 2020 guide, we searched for articles that studied the relationship among sarcopenia and metabolic risk factors in adults over 65 years of age, published between 2012 and 2022 in four databases: PubMed, Web of Science, Cochrane Plus, and CINAHL. A total of 370 articles were identified in the initial search, of which 13 articles were selected for inclusion in this review. It was observed that metabolic risk factors such as Body Mass Index, systolic and diastolic blood pressure, glucose, cholesterol, or triglycerides had a significant association with sarcopenia. There is evidence of the association of different metabolic risk factors with sarcopenia in adults over 65 years of age, so it is necessary to carry out studies that investigate different strategies that reduce the appearance of sarcopenia, and with it, the incidence of metabolic diseases.

## 1. Introduction

The world population is aging dramatically. Since 1980, the number of people aged 60 years or more has reached 810 million and is expected to increase to 2 billion by 2050, with 22% of the total population predicted to be over 60 years of age and 5% over 80 years of age by that year [1]. This aging process has led to more and more people becoming susceptible to the various diseases associated with this phase of life [2], and in particular, it is responsible for numerous changes in body composition, including the loss of skeletal muscle mass [3]. To define this phenomenon, the name sarcopenia was attributed, which has an increasing impact among those over 65 years of age and is considered a frequent cause of mortality in this age group [4]. As a result, since 2016, it has been considered by the World Health Organization (WHO) as a disease [5].

The concept of sarcopenia was first proposed by Dr. Irwin Rosenberg in 1989 [6], but it was the European Working Group on Sarcopenia in Older People (EWGSOP) that developed a definition that was an important change, since it included the muscle function in the previous definitions based exclusively on the detection of low muscle mass [7]. Similarly, in 2018, the EWGSOP2 used low muscle strength as a core element of sarcopenia in its definition and reported that low muscle strength, low muscle quality/quantity, and poor physical performance are hallmarks of severe sarcopenia [7,8]. This loss of muscle mass begins to occur at the age of 30 years which, together with poor physical performance, can result in a person loosing 10% of his or her muscle mass by the age of 50 years. On average, muscle mass loss is approximately 5 kg every 10 years after the age of 40 [9]. Therefore, the prevalence of sarcopenia varies between 1% and 50% in the elderly [10] and it is estimated that 5% to 13% of people aged 60–70 years suffer from sarcopenia and 11% to 50% in older people aged 80 years or more [11].

Sarcopenia is a complex disease involving genetic and environmental factors, and its occurrence involves many mechanisms and risk factors, among which metabolic risk factors stand out, since sarcopenia is closely related to other diseases such as cardiovascular and metabolic diseases [12], with an increased risk of falls, fractures, disability, and even mortality, and with it, an increased social, medical and economic burden [13]. Metabolic diseases refer to a series of signs and symptoms that include risk factors such as hypertension, dyslipidemia, obesity, or hyperglycemia, with insulin resistance being the most common [14]. Previous studies have shown that the reduction in skeletal muscle mass increases insulin resistance, which is related to the development of metabolic diseases [15,16], while an increase in body mass could lead to an improved insulin sensitivity [17]. It has also been observed that sarcopenia causes atherosclerosis and leads to high blood pressure [18]. 

Considering that during the aging process, changes in body composition, an increase in fat mass, and a reduction in skeletal muscle may increase the risk of metabolic diseases—and with it, a functional deterioration—the aim of this work was to analyze the relationship among different metabolic risk factors and sarcopenia in people over 65 years of age.

## 2. Materials and Methods

The present study was conducted following PRISMA 2020 guidelines [19,20] and the pre-specified protocol was registered in PROSPERO (CRD42023391415).

### 2.1. Sources of Information and Search Strategy

Data collection was performed in the months of November and December 2022 in the following databases: Pubmed, Web of Science, CINAHL, and Cochrane. Different keywords were used for the search, as well as the Boolean operators “AND” and “OR”, resulting in the following search string: (“Sarcopenia”) AND (“Metabolic Diseases” OR “Thesaurismosis” OR “Thesaurismoses” OR “Metabolic Disease” OR “Disease, Metabolic” OR “Diseases, Metabolic”) AND (“older adults” OR “older women” OR “older men” OR “elderly” OR “seniors” OR “aging”).

### 2.2. Eligibility Criteria

The inclusion criteria for this systematic review and meta-analysis were as follows: cross-sectional articles studying the association of metabolic risk factors with sarcopenia; conducted in participants older than 65 years of age. The search was limited to studies within the last 10 years and with human participants. Articles that did not report or measure metabolic risk factors in subjects with or without sarcopenia, as well as studies that did not present a clear definition or criteria for the diagnosis of sarcopenia, were excluded. 

### 2.3. Study Selection and Data Extraction

First, duplicate articles and those without abstracts were discarded. Two independent authors (M.C.C.-F. and F.H.-C.) selected the titles and abstracts based on the eligibility criteria and two other authors (A.A.-A and M.M.C.F.) reviewed the full texts. Finally, discrepancies were resolved by consensus with a third author (D.F.A.-R). Data on authors, year of publication, country, setting, study design, sample size, sex, definition of sarcopenia, and adjustments were included.

### 2.4. Assessment of Methodological Quality

For the assessment of the methodological quality of the studies included in this review, the critical appraisal tool of the Joanna Briggs Institute (JBI), frequently used in systematic reviews, was used [21]. The JBI is an 8-item scale, with the following items: (i) Were the criteria for inclusion in the sample clearly defined?; (ii) Were the study subjects and the setting described in detail?; (iii) Was the exposure measured in a valid and reliable way?; (iv) Were objective, standard criteria used for measurement of the condition?; (v) Were confounding factors identified?; (vi) Were strategies to deal with confounding factors stated?; (vii) Were the outcomes measured in a valid and reliable way?; (viii) Was appropriate statistical analysis used? The score obtained with this tool provides a maximum of 8 points for cross-sectional studies, which indicates high study quality, and a score ≥ 5 is considered good quality [22].

### 2.5. Analytic Decisions for Meta-Analysis

Data analyses were performed using the Comprehensive Meta-Analysis Software (CMA-V4). A random-effects meta-analysis was performed to calculate the association among metabolic risk factors and sarcopenia with 95% confidence intervals (95% CI) by measuring the Odds ratio (OR); values less than 1 indicate a negative association, values greater than 1 indicate a positive association, and values equal to 1 indicate no association. 

The results of the meta-analysis are presented through the forest plot, recording the first author, the date of publication, the individual effects, the 95% CI, and the overall effect with the 95% CI, as well as the *p*-value associated with the statistic. A subgroup analysis by sex was performed. To address possible publication bias, graphical analyses were performed using funnel plots and their distribution.

## 3. Results

### 3.1. Selection of the Studies

**Figure 1:**
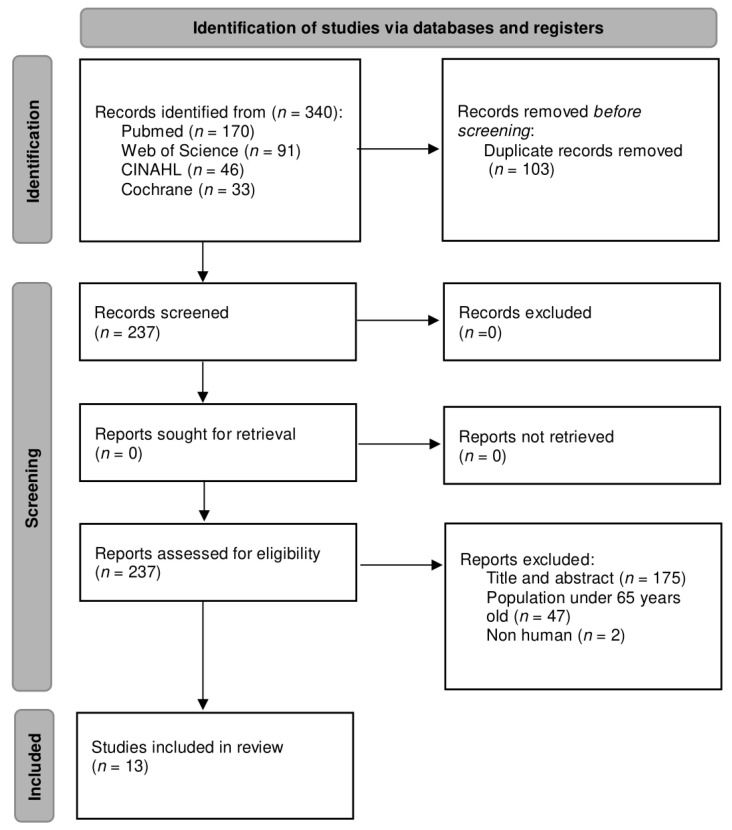
Flow diagram of the study selection process.

A complete search was carried out in different databases, resulting in a total of 340 articles. Subsequently, a filtering for duplicate articles was performed, leaving a total of 237 unique articles. These articles were then examined considering the title and abstract and the eligibility criteria; only 13 articles were included [23,24,25,26,27,28,29,30,31,32,33,34,35], while the other 224 articles were excluded. The flow diagram of the study selection based on the PRISMA statement [36] is presented in Figure 1.

### 3.2. Methodological Quality

The methodological quality of the studies was assessed using the JBI scale. The scores obtained from the 13 articles were calculated manually and all the scores were ≥6 points, considered as good quality, with 5 of the studies [25,27,29,32,35] obtaining the maximum score (8 points), which indicates an excellent quality. The summary of the scores of the studies included in this systematic review and meta-analysis is presented in Table 1.

### 3.3. Characteristics of the Studies

The articles selected in this systematic review and meta-analysis were cross-sectional studies published in South Korea [23,24,25,26,30,31,32], Germany [27,33], Japan [28], and Brazil [29] during the period between 2013 and 2022 (2013 [23,26,30], 2014 [28,32], 2015 [29,31], 2016 [24,27], 2017 [25], 2018 [35], 2021 [34], and 2022 [33]). All articles were written in English. 

A total of 1,480,466 people participated in the selected studies, of whom 452,501 had sarcopenia and 867,991 did not have sarcopenia. The sample size of the 13 articles included in this systematic review with meta-analysis varied from 173 [29] to 1,457,413 individuals [24]. Four articles enrolled only men [29,30,32,35], one article included only women [25], and eight articles incorporated both sexes in their studies [23,24,26,27,28,31,33,34]. All details of the articles selected in this review are presented in Table 2.

### 3.4. Definition of Sarcopenia

Dual-energy X-ray absorptiometry is a low radiation technique that is frequently used for the evaluation of body composition [37]. Most of the included studies use this assessment method and define sarcopenia as the existence of appendicular skeletal mass/weight of <1 standard deviation below the sex-specific mean considering a young reference group [23,24,25,26,27,30,32,35], while the article by Moon et al. [31] defines it as the existence of appendicular skeletal mass/weight of <2 standard deviation below the sex-specific mean for young adults. Similarly, Park et al. [34] define it as the existence of appendicular skeletal mass/height^2^ and the article by Buchmann et al. [33] measure through dual-energy X-ray absorptiometry body composition and established cut-off points identified in the Foundation for the National Institutes of Health (FNIH) Sarcopenia Project.

Furthermore, several studies consider other outcomes measures for the assessment and diagnosis of sarcopenia such as muscle strength assessed through hand grip strength using a dynamometer [27,28,29], skeletal muscle mass and fat mass through bioelectrical impedance [28], appendicular skeletal muscle mass derived from the sum of the muscle mass of the four limbs [28], low muscle mass defined as relative appendicular skeletal muscle mass measured through the equation: appendicular lean mass/height^2^ [29], and finally, physical performance assessed by habitual gait speed, a test consisting of walking straight for 11 m at their usual speed [28].

### 3.5. Associations of Metabolic Risk Factors with Sarcopenia

In all the selected studies, the relationship among different metabolic risk factors and sarcopenia was analyzed, and in all of them, a significant association could be observed. In two of the selected articles [25,35], the prevalence of metabolic syndrome was higher in the group with sarcopenia, and in the study by Kang et al. [25], the prevalence was even higher in those participants with sarcopenic obesity (OR: 6.26, CI: 5.10–7.07), conceiving the loss of muscle mass as an independent risk factor that could cause metabolic syndrome.

In two studies [23,29], the association of bone mineral density with sarcopenia was tested and in both it was observed that people with sarcopenia had a low level of calcium; moreover, in the study of Lee et al. [23], a significant association between sarcopenia and vitamin D insufficiency (25(OH)D, (ng/mL). Beta 0.065, *p* = 0.003) was found. Likewise, Chung et al. [26] found that the prevalence of vitamin D deficiency and metabolic syndrome was higher in the sarcopenic obese group (25−hydroxyvitamin D levels 18.50 ± 7.10). With respect to other metabolic risk factors, two studies [26,32] showed that people with sarcopenia presented higher levels of insulin resistance (12.30 ± 6.20). Five articles [24,27,30,31,32] observed that fat mass, BMI, and waist circumference were significantly higher in subjects with sarcopenia. Moreover, within these five studies, three [27,31,32] reported that sarcopenia is significantly associated with an increase in triglycerides; three articles [24,27,30] analyzed the possible relationship of HDL cholesterol in patients with sarcopenia and out of these three, two studies [27,30] confirmed the relation, while the article by Choi et al. [24] found a lower HDL cholesterol in the female group with sarcopenia (males, x^2^ = 27.36, *p* < 0.010 vs. females, (x^2^ = 13.75, *p* < 0.050). 

Regarding blood pressure, three studies [24,27,31] analyzed its association with sarcopenia; two of them [24,31] showed a relationship only with systolic blood pressure, while one article [27] reported that both, systolic, and diastolic blood pressure were higher in people with sarcopenia. In relation to muscle mass and lean mass, two studies [28,33] found that people with sarcopenia showed lower muscle mass; in addition, Ishii et al. [28] measured grip strength and found that it was also lower in people with the disease (27.50 ± 4.30 vs. 36.00 ± 5.30). Concerning glucose, four articles [24,27,27,30,31] studied the relationship of glucose in people with sarcopenia and three of them [27,30,31] observed that fasting glucose had a significant negative correlation with the relationship between appendicular skeletal muscle mass and body weight. However, the study by Choi et al. [24] showed that in the female group, women with sarcopenic obesity had fasting blood glucose within normal values.

Finally, Park et al. [34] addressed an additional EWGSOP criterion for the diagnosis of sarcopenia, namely physical performance, and found that people with sarcopenia who were moderately active physically had lower body weight, body fat mass, and body fat percentage, and higher free fat mass and muscle mass than in people with sarcopenia with low physical activity. They also observed that the incidence of metabolic syndrome was significantly lower in those with higher active physical performance (OR 0.47, 95%CI: 0.30–0.75).

### 3.6. Metabolic Syndrome and Sarcopenia

**Figure 2:**
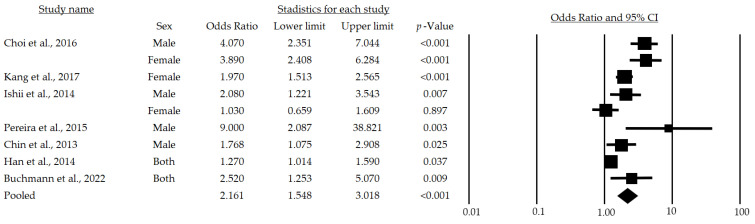
Forest plot of the association of metabolic syndrome and sarcopenia in both sexes [24,25,28,29,30,32,33]. The black box represents the point estimate for the respective study, while the size of the box represents the population size, and the horizontal line is the 95% CI. The diamond-shaped figure represents the estimated point of the mean odds ratio.

Figure 2 provides the results of the meta-analysis of seven studies. The lowest association statistic reported by the included studies was an OR of 1.03 [28] and the highest was 9.0 [29]. The randomized model provided an OR of 2.16 and a 95% (95%CI: 1.54–3.01), demonstrating a positive association among metabolic risk factors and sarcopenia in adults over 65 years of age.

**Figure 3:**
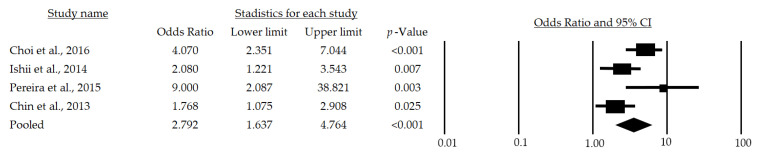
Forest plot of the association of metabolic syndrome and sarcopenia in men [24,28,29,30]. The black box represents the point estimate for the respective study, while the size of the box represents the population size, and the horizontal line is the 95% CI. The diamond-shaped figure represents the estimated point of the mean odds ratio.

After a subgroup analysis, a significant OR of 2.79 (95%CI: 1.637–4.764; *p* < 0.001) was found (Figure 3), which indicates that the chance of developing sarcopenia in adult men over 65 years of age is 179% higher in those at metabolic risk than in those who are not at risk.

**Figure 4:**
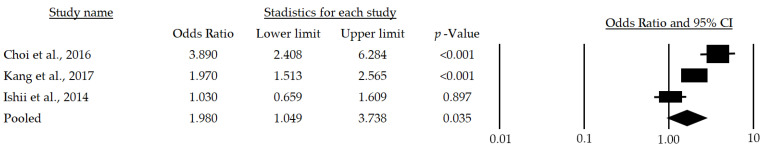
Forest plot of the association of metabolic syndrome and sarcopenia in women [24,25,28]. The black box represents the point estimate for the respective study, while the size of the box represents the population size, and the horizontal line is the 95% CI. The diamond-shaped figure represents the estimated point of the mean odds ratio.

Meanwhile, women over 65 years of age who have metabolic risk factors have a 98% greater chance of developing sarcopenia (OR: 1.98; 95%CI: 1.049–3.738; *p* = 0.035) (Figure 4).

To handle the predominance of the Korean population, a meta-regression was used, from which it was possible to adjust for the population as a possible confounding factor to evaluate whether it could influence the effect size; as a result of the regression, the coefficient for this covariate was obtained and was close to zero (0.02), which discards its influence in the estimation of the effect size. Likewise, the coefficient of determination (R-squared) was equal to zero, and the estimated effect size in OR was 1.1 with a pe of 0.99, thus indicating that the population covariate included in the model is not significantly affecting the results.

A sensitivity analysis was carried out excluding studies that contained duplicate individuals or data and then comparing the results against those obtained from the complete meta-analysis. Therefore, it was possible to evidence that there were no differences in this regard. 

### 3.7. Publication Bias

The analysis was performed using the funnel plot, including all the articles of the meta-analysis, which revealed an expected publication bias, as there were articles that diverged with respect to the measure of association and modified the OR by 25%. However, when the analysis of subgroups by sex was performed, the heterogeneity was reduced, which allowed a greater symmetry in the distribution to be evidenced, remaining within the confidence interval.

## 4. Discussion

This systematic review with meta-analysis, performed with the aim of determining the relationship among metabolic risk factors and sarcopenia in persons over 65 years of age, considered 13 studies [23,24,25,26,27,28,29,30,31,32,33,34,35] that met the eligibility criteria. After reviewing these articles, it was found that the metabolic risk factors analyzed in the different studies were associated with sarcopenia in people over 65 years of age and that metabolic syndrome is, indeed, a risk factor for sarcopenia (mean OR: 2.161, CI: 1.548–3.018, *p* < 0.001), regardless of sex (Male, mean OR: 2.792, CI: 1.637–4.764, *p* < 0.001; Female, mean OR: 1.980, CI: 1.049–3.738, *p* = 0.035).

Sarcopenia is very common among older people because several changes occur with age, such as a significant decrease in muscle mass, strength, and physical activity [38]—factors that are recognized by the EWGSOP as criteria for the diagnosis of sarcopenia [7,8]. It is important to note that, in most of the selected studies, the definition of sarcopenia was very similar and the most used measurement techniques were DXA and BIA. These characteristics strengthen the associations observed and thus increase the homogeneity of the review performed.

The loss of muscle mass is related to age, which at the same time is associated with physical disability, metabolic alterations, and an increased mortality [39]. Regarding metabolic risk factors, in the present review it was found in 3 of the 13 studies [24,27,31] that arterial pressure is associated with a decrease in muscle mass. These findings are in line with another previous study [40] that reflects that low muscle mass is associated with cardiovascular risk factors such as arterial stiffness, which raises the additive effects of this low muscle mass on blood pressure, and with it, a higher prevalence of hypertension in people with sarcopenia. Likewise, in two studies in this review [26,32], it was observed that participants with sarcopenia had higher levels of insulin resistance. This significant relationship may be explained, as having low muscle mass may mean that a person is more susceptible to insulin resistance [41]. Moreover, with increasing age, the ability of muscle mitochondria to catalyze fatty acid metabolism is reduced, resulting in an acceleration of insulin resistance associated with obesity and glucose metabolism disorders [42]. Regarding the relationship between sarcopenia and BMD, two articles [23,29] confirmed this association, as well as a previous study [43] that identified a correlation between sarcopenia assessed by the Relative Skeletal Muscle Mass Index and BMD in postmenopausal women. This relationship may be due to the production of lower mechanical stimulation and proinflammatory cytokines underlying sarcopenia [44,45].

Additionally, due to the decrease in muscle mass associated with sarcopenia, the metabolic rate is reduced, which can lead to an increase in body fat, and with it, metabolic disorders, and functional disability [46]. In agreement with this statement, five studies included in the present review [24,27,30,31,32] found that both men and women with sarcopenia had higher levels of fat mass, BMI, and waist circumference. In the case of women, the higher values may be explained by the reduction in estrogen levels produced by menopause that cause an increase in body weight and changes in fat accumulation in subcutaneous deposits [47], while in men older than 65 years, the reduction in testosterone levels are intimately related to sarcopenia and in turn to lower muscle strength, lower physical performance, and consequently, a higher risk of falls [48].

Decreased muscle strength leads to increased incidence of both sarcopenia and sarcopenic obesity and the occurrence of cardiovascular and metabolic diseases [49]. Previous studies have analyzed the relationship among low muscle strength and metabolic risk factors and have concluded that grip strength has been correlated with metabolic syndrome [50] and hypertension [51]. Consistent with these studies, one article selected in this review [28] found that participants with sarcopenia had lower grip strength. Moreover, two of the selected articles [23,26] observed an association between sarcopenia and vitamin D, which is a key factor in calcium regulation and bone metabolism [52]. This association may be because vitamin D deficiency could affect muscle function and lead to reduced muscle strength and balance, thus increasing the risk of falls and loss of autonomy in the elderly [53]. In addition, two articles [27,30] showed higher HDL cholesterol in patients with sarcopenia, although another included article [24] showed that females with sarcopenia had lower HDL cholesterol levels. Consistent with our results is the study by Lee et al. [54], which found no significant associations between grip strength and cholesterol in people of both sexes and aged over 53 years.

The findings found in this review are intimately related to aging, considering the age of the participants (older than 65 years). As could be observed in one of the selected studies [34], in addition to the aging process, reduced physical exercise is another diagnostic criterion for sarcopenia that may contribute to the development of metabolic risk factors [55]. Previous studies have shown that physical inactivity causes the development of sarcopenia [56,57]; moreover, a systematic review and meta-analysis [58] proved the beneficial effects of physical activity on sarcopenia in older people. Therefore, experimental studies analyzing the effects of different methods of physical exercise on metabolic factors associated with sarcopenia are needed. 

Finally, the meta-analysis performed suggests that metabolic syndrome or its risk factors (hypertriglyceridemia, increased waist circumference, low HDL cholesterol, arterial hypertension, and fasting blood glucose) [59] are a risk for presenting sarcopenia (mean OR: 2.161, CI: 1.548–3.018, *p* < 0.001), being higher for men (mean OR: 2.792, CI: 1.637–4.764, *p* < 0.001) than for women (mean OR: 1.980, CI: 1.049–3.738, *p* = 0.035). 

This systematic review has several strengths such as having included studies with good methodological quality (measured by JBI); the selected articles mainly had a large sample size and in total represent 1,480,707 persons older than 65 years, including both sexes, which may be a representative sample; most of the studies used the same definition of sarcopenia, which gives consistency to the categorization of the studies and their comparison. However, it is possible to identify a geographical bias, as the articles included are from Europe, Africa, and Asia without including research conducted in the Americas or Australia, which possibly limits the generalizability of the results of this review.

## 5. Conclusions

Sarcopenia has become an important health condition associated with aging. The present systematic review with meta-analysis revealed that there is a significant association among the metabolic risk factors studied (BMI, SBP, DBP, DBP, TG, fasting glucose, HOMA-IR, and HDL) and sarcopenia in persons older than 65 years in both sexes, although the risk is higher for men than for women. Finally, the association between sarcopenia and metabolic syndrome in older adults could benefit from cohort studies as it would allow the assessment of risk during follow-up and the influence of time of exposure to the factors as well as studies with detailed data stratified by types of sarcopenias.

## Figures and Tables

**Table 1:** Joanna Briggs Institute (JBI) scores for cross-sectional studies.

Study	Item 1	Item 2	Item 3	Item 4	Item 5	Item 6	Item 7	Item 8
Lee et al., 2013 [23]	+	+	+	+	-	-	+	+
Choi et al., 2016 [24]	+	-	+	+	+	+	+	+
Kang et al., 2017 [25]	+	+	+	+	+	+	+	+
Chung et al., 2013 [26]	+	+	+	+	-	-	+	+
Buchmann et al., 2016 [27]	+	+	+	+	+	+	+	+
Ishii et al., 2014 [28]	+	+	+	+	-	-	+	+
Pereira et al., 2015 [29]	+	+	+	+	+	+	+	+
Chin et al., 2013 [30]	+	+	+	+	-	-	+	+
Moon et al., 2015 [31]	+	+	+	+	-	-	+	+
Han et al., 2014 [32]	+	+	+	+	+	+	+	+
Buchmann et al., 2022 [33]	+	+	+	-	+	+	+	+
Park et al., 2021 [34]	+	+	+	+	-	-	+	+
Sung et al., 2018 [35]	+	+	+	+	+	+	+	+

Item 1: Were the criteria for inclusion in the sample clearly defined?; Item 2: Were the study subjects and the setting described in detail?; Item 3: Was the exposure measured in a valid and reliable way?; Item 4: Were objective, standard criteria used for measurement of the condition?; Item 5: Were confounding factors identified?; Item 6: Were strategies to deal with confounding factors stated?; Item 7: Were the outcomes measured in a valid and reliable way?; Item 8: Was appropriate statistical analysis used?; +: Yes; -: No.

**Table 2:** Characteristics of the included studies (*n* = 14).

Author (Year)	Country, Setting	Study Design	Sample Size (with/without Sarcopenia)	Sex (%)	Definition of Sarcopenia	Adjustments
Lee et al., 2013 [23]	South Korea, Social	Cross sectional study	T: 3482 (1453/2029)	M: 45.83 F: 54.17	ASM was assessed by DXA; ASM/Wt was as <1 SD below the sex specific mean for a young reference group	BMI, fasting glucose, HOMA-IR
Choi et al., 2016 [24]	South Korea, Social	Cross sectional study	T: 1,457,413 (444,426/854,420)	M: 44.56 F: 55.44	ASM was assessed by DXA; ASM/Wt was as <1 SD below the sex specific mean for a young reference group	BMI, fasting glucose, SBP, DBP, TG, HDL-C, total cholesterol
Kang et al., 2017 [25]	South Korea, Social	Cross sectional study	T: 4183 (2771/1412)	F: 100	ASM/Wt was less than 1 standard deviation (SD) below the mean of the reference group	BMI, fasting glucose, SBP, DBP, TG, HDL-C, total cholesterol
Chung et al., 2013 [26]	South Korea, Social	Cross sectional study	T: 2943 (1248/1665)	M: 42.47 F: 57.53	ASM/Wt was less than 1 SD below a sample of healthy adults aged	BMI, fasting glucose, SBP, DBP, TG, HOMA-IR, HDL-C, LDL-C, total cholesterol
Buchmann et al., 2016 [27]	Germany Community-dwelling	Cross sectional study	T: 1402 (280/1122)	M: 51.07 F: 48.93	Body composition: DXA; hand grip strength: Dynamometer	BMI, fasting glucose, TG, HOMA-IR, HDL-C
Ishii et al., 2014 [28]	Japan Social	Cross sectional study	T: 1971 (359/1612)	M: 50.43 F: 49.57	Muscle mass: BIA; ASM: the sum of the muscle mass of the four limbs; muscle strength: dynamometer; physical performance: usual gait speed.	BMI
Pereira et al., 2015 [29]	Brazil Social	Cross sectional study	T: 173 (20/153)	M:100	Low muscle mass: RASM (aLM/height^2^); muscle strength: handgrip strength	BMI
Chin et al., 2013 [30]	South Korea, Social	Cross sectional study	T: 1076 (176/900)	M:100	ASM by DXA: ASM/Wt was less than 1 SD below a sample of healthy adults aged	BMI, fasting glucose, TG, HOMA-IR, HDL-C, LDL-C, total cholesterol
Moon et al., 2015 [31]	South Korea, Social	Cross sectional study	T: 674 (35/639)	M: 47.16 F: 52.84	ASM/Wt was less than 2 SD below a sample of healthy adults aged (DXA)	BMI, fasting glucose, SBP, DBP, TG, HDL-C
Han et al., 2014 [32]	South Korea, Social	Cross sectional study	T: 1502 (459/1043)	M: 100	ASM/Wt, 1 SD below the mean of a sample of healthy adults	BMI, fasting glucose, SBP, DBP, TG, HOMA-IR, HDL-C, total cholesterol
Buchmann et al., 2022 [33]	Germany, community-dwelling	Cross sectional study	T: 1377	M: 48.9 F: 51.1	Body composition: DXA	BMI, HDL, glucose level, CRP
Park et al., 2021 [34]	South Korea, Social	Cross sectional study	3573 (1135/2438)	M: 42.91 F: 57.09	ASM/height^2^, by DXA	BMI, fasting glucose, TC, TG, LDL-C and HDL-C
Sung et al., 2018 [35]	South Korea, Social	Cross sectional study	697 (139/558)	M: 100	ASM/weight, by DXA, 1 SD below the mean of a sample of healthy adults	BMI, TG, HDL, fasting blood sugar

T: Total sample size; M: Male; F: Female; aLM: appendicular lean mass; ASM: Appendicular skeletal muscle mass; ASM/Wt: appendicular skeletal muscle mass/weight; BIA: bioelectrical impedance analyzer; BMI: body mass index; CRP: C-reactive protein; DBP: diastolic blood pressure; DXA: dual-energy X-ray absorptiometry; FBG: fasting blood glucose; HDL-C: high-density lipoprotein-cholesterol; HOMA-IR: homeostatic model assessment of insulin resistance; LDL-C: a low-density lipoprotein-cholesterol; SD: standard deviation; SBP: systolic blood pressure; TC: total cholesterol; TG: serum triglyceride; RASM: relative appendicular skeletal muscle mass.

## Data Availability

All available data can be obtained by contacting the corresponding author.
